# Complete genome sequence of *Thiomicrospira* sp. strain V2501 isolated from 250 m below the ground level in Horonobe, Hokkaido, Japan

**DOI:** 10.1128/mra.00108-24

**Published:** 2024-10-22

**Authors:** Akio Ueno, Kiyoshi Sato, Shuji Tamamura, Takuma Murakami, Hidenori Inomata, Satoshi Tamazawa, Yuki Amano, Kazuya Miyakawa, Takeshi Naganuma, Toshifumi Igarashi

**Affiliations:** 1 Horonobe Research Institute for the Subsurface Environment (H-RISE), Northern Advancement Center for Science and Technology (NOASTEC), Hokkaido, Japan; 2 Horonobe Underground Research Center, Japan Atomic Energy Agency (JAEA), Hokkaido, Japan; 3 Graduate School of Integrated Sciences for Life, Hiroshima University, Hiroshima, Japan; 4 Faculty of Engineering, Hokkaido University, Hokkaido, Japan; 5 National Institute of Technology, Asahikawa College (KOSEN), Hokkaido, Japan; SUNY College of Environmental Science and Forestry, Syracuse, New York, USA

**Keywords:** subsurface environment, chemolithoautotrophy, *Thiomicrospira* sp.

## Abstract

A thiosulfate-oxidizing bacterium, *Thiomicrospira* sp. strain V2501, was isolated from groundwater collected in a terrestrial deep subsurface environment. This strain was capable of chemolithoautotrophic growth on CO_2_ and thiosulfate. Here, we report the 2,240,851 bp complete genome sequence of strain V2501.

## ANNOUNCEMENT

The genus *Thiomicrospira* (*Tms*.) (*Gammaproteobacteria*, *Thiotrichales*, and *Piscirickettsiaceae*) was originally described by Kuenen and Veldkamp (1). Currently, six *Tms*. species have been validated (2) and isolated from soda and hypersaline lakes (3–5), estuarine mud (1), and the gill tissue of the marine bivalves (6). We isolated *Thiomicrospira* sp. strain V2501 and presented its complete genome sequence.

*Thiomicrospira* sp. strain V2501 was obtained from groundwater collected on 15 December 2009 from the borehole 09-V250-M02 at 250 m below ground level of the Horonobe Underground Research Laboratory, Hokkaido, Japan, by colony isolation at 30°C. The sampling site was described previously (7). The enrichment cultivation was performed aerobically in DSMZ 484 medium with slight modifications (NaCl concentration was changed from 25.0 to 23.4 g/L and 0.5 g/L of NaHCO_3_, pH 8.5–9.0) at 30°C until the medium turned turbid. Strain V2501 was isolated from single colonies on agar plates (15.0 g/L of agar) of the same medium. Purity was confirmed using microscopic observation and direct sequencing of the 16S rRNA gene (at least 10 reads) as described previously (8). Strain V2501 grew chemolithoautotrophically under aerobic conditions.

Strain V2501 was propagated aerobically in 100 mL of the same medium at 30°C and was harvested by centrifugation. Genomic DNA was extracted using a Genomic-tip 20/G DNA purification kit (Qiagen, Hilden, Germany), following the manufacturer’s protocol. The quality of the genomic DNA was assessed using a 5200 Fragment Analyzer System and an Agilent HS Genomic DNA50 kb kit (Agilent Technologies, CA, USA). The genomic DNA was sheared into 10–20-kb fragments using a g-TUBE device (Covaris). A library was constructed using an SMRTbell Template Prep Kit Version 2.0 (PacBio, Menlo Park, CA, USA). Polymerase complexes were formed using the Binding Kit 2.2 (PacBio, Menlo Park, CA, USA). These complexes were sequenced using the PacBio Sequel lle System (Menlo Park, CA, USA). The adapter overhang sequence was eliminated to generate sub-reads using SMRT Link (version 10.1.0. 119528) with default parameters. High-fidelity (HiFi) reads (34,433 reads) were acquired by eliminating reads with average quality values < 20. HiFi reads shorter than 1,000 bp were removed using Filtlong version 0.2.0. HiFi reads exceeding 1,000 bp were *de novo* assembled using Flye version 2.9 with default parameters (9). The assembly graph was visualized using Bandage (version 0.8.1) (10), indicating that the assembled genome was circular. The genome coverage was 111.6×, and the *N*_50_ value was 2,240,851 bp. The integrity of the assembled genome data was assessed using CheckM version 1.2.0 (11). Annotation was performed using Prokka version 1.14.5 for coding sequence, rRNAs, and tRNAs (12).

**Fig 1:**
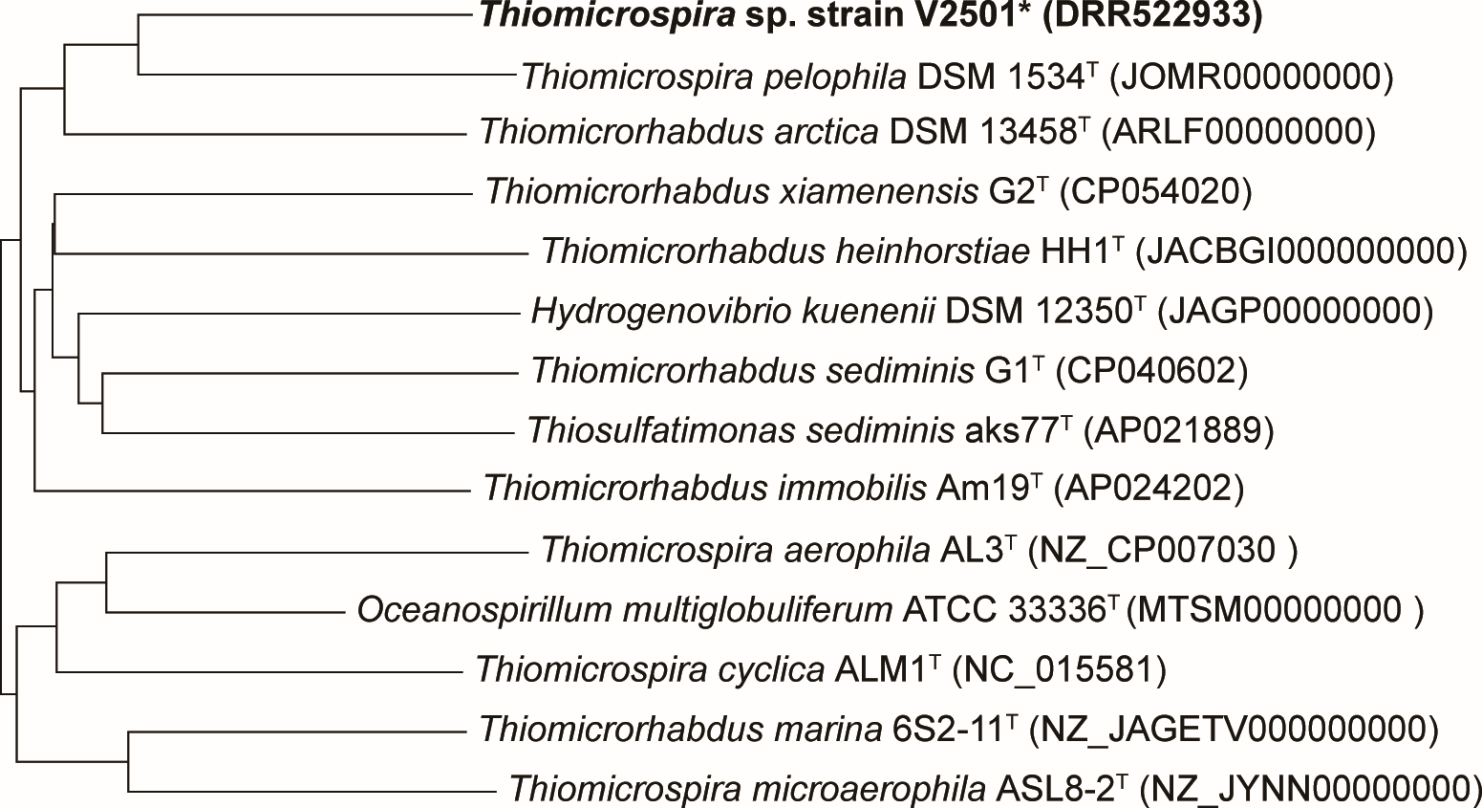
Phylogenomic tree of *Thiomicrospira* sp. strain V2501 (marked with an asterisk) and related strains was obtained using the Type (Strain) Genome Server platform (https://tygs.dsmz.de/) (13). The tree was inferred with FastME 2.1.6.1 (16) using GBDP (Genome Blast Distance Phylogeny) distances calculated from genome sequences. The accession numbers of the genome sequences are shown in parentheses.

The genome sequencing data and statistics are listed in Table 1. A phylogenomic tree constructed using TYGS (13) indicated that strain V2501 was most closely related to *Tms. pelophila* DSM 1534^T^ (Fig. 1), and the OrthoANIu value was 83.2%, which was below the species cut-off value (95% ± 0.5%) (Table 1) (14, 15).

**TABLE 1:** Summary of sequence data and genome features of *Thiomicrospira* sp. strain V2501

Parameters	Data
Genome features and statistics	
No. of HiFi reads	34,433
Average length (bp)	7,260.1
Total length (bp)	249,986,951
After genome assembly	
Genome length (bp)	2,240,851
No. of contigs	1 (a single circular contig)
Genome coverage	111.6×
*N*_50_ (bp)	2,240,851
G + C content (mol%)	45.1
No. of coding sequence	2,135
No. of RNA genes	9
No. of 5S RNA genes	3
No. of 16S RNA genes	3
No. of 23S RNA genes	3
No. of tRNA genes	43
No. of CRISPR loci	3
Average nucleotide identity values (%) between strain V2501 and	
*Thiomicrospira pelophila*	83.22
*Thiomicrospira aerophila*	71.81
*Thiomicrospira cyclica*	71.87
*Thiomicrospira microaerophila*	72.78

## Data Availability

All data were deposited in NCBI/ENA/DDBJ under accession numbers AB634592 for the 16S rRNA gene, and DRA017671/DRR522933, SAMD00726318, and PRJDB17264 for Sequence Read Archive (DRA), BioSample, and BioProject, respectively.
